# Chronic diseases: 2010–2011 progress of the unit for chronic patients and evidence-based medicine of Donostia Hospital/Patologías crónicas: evolución 2010–2011 de la unidad de crónicos y medicina basada en la evidencia del Hospital Donostia

**Published:** 2012-05-29

**Authors:** Joxe Artetxe, Kepa Aranegi, Mª Isabel Huertas, Jose Emparanza, Iñaki Dorronsoro, Elena Suquía

**Affiliations:** Unit for Chronic Patients and Evidence-Based Medicine (EBM), Donostia Hospital, Donostia, Basque Country, Spain; Unit for Chronic Patients and EBM, Donostia Hospital, Donostia, Basque Country, Spain; Unit for Chronic Patients and EBM, Donostia Hospital, Donostia, Basque Country, Spain; Donostia Hospital, Basque Country, Spain; Donostia Hospital, Basque Country, Spain; Donostia Hospital, Donostia, Basque Country, Spain

**Keywords:** heart failure, pulmonary disease, chronic obstructive, frail elderly, nursing home, self care, fallo cardiaco, enfermedad pulmonar obstructiva crónica, paciente frágil, residencia gerontológica, autocuidado

## Introduction

Donostia hospital has a catchment population of 700,000 covering the province of Gipuzkoa (Basque Country, Spain).

In 2009, the management of chronic patients was identified as a strategic area for the hospital, a specific structure being established in 2011 for the study of chronicity and improvement in its management: the unit for chronic and convalescent patients and evidence-based medicine (EBM). The core of the care provided in this unit has a common element: the management of processes based on EBM.

Since 2005, Donostia Hospital has being carrying out studies to identify patients with a history of multiple admissions, together with small-scale interventions (monitoring of heart failure and COPD). This approach has been found to be effective in decreasing the number of hospital bed days and attendances to the Emergency Department, while the efficiency of telemedicine has not yet been established. The following step to be considered was the extension of these interventions to the general population.

The objectives set for 2010 were: to establish a large-scale intervention for people with history of multiple admissions; to set up an intervention involving nursing homes for the elderly in Gipuzkoa; to ensure continuous contact with primary care; and to progressively involve users and caregivers in the management and monitoring of user’s medical conditions.

## Description of the projects

We describe below four of the studies carried out by the Unit for Chronic Patients and EBM at Donostia hospital.

### EMAI project (hospital-based individualised multifaceted care strategy)

#### Objectives

- To assess the impact of an intensive multifaceted care programme on the quality of care perceived by the frail patient.
- To assess the impact of this programme on compliance with treatment.
- To assess its impact on the number of attendances to the emergency department and readmissions.

#### Methods

Design: It was a before and after study of cohort of patients with a history of multiple admissions due to heart failure or COPD and with more than one admission related to this condition in the year prior to their inclusion.

Study population: A total of 242 patients with a history of multiple admissions due to heart failure or COPD seen at the Unit for Chronic Patients and EBM at Donostia Hospital.

Description of the EMAI Project: It involves the identification of frail patients and labelling their electronic record, provision of personalised information, assignment to a referral doctor and to a case manager nurse, provision of a direct number for advice throughout the day, contact with the GP, agreement on goals, monthly check-ups, monthly telephone call between the check-ups, and admission where necessary through their assigned doctors, without going through the Emergency Department.

#### Results

By 31st December 2010, there were 242 participants of whom 132 had both conditions, 216 people being coded as having heart failure and 158 with COPD.

- A 67% decrease in hospital bed days (3000 fewer hospital bed days in the study year).
- A 77% decrease in the number of attendances to the Emergency Department.
- Compliance with treatment of over 90%.

#### Perceived quality

Focus groups: very positive assessment of the organisation of discharge, information provided, coordination between centres and services, treatment and follow-up in doctor’s offices and by telephone.

### Controlled clinical trial of care for geriatric patients in nursing homes

#### Primary objectives

To assess:

- the efficacy of a multidisciplinary care programme that includes telemonitoring, measured in terms of the number of admissions.
- the appropriateness and acceptability of a proposed agreement concerning the planning of care in the opinion of all those involved.

#### Secondary objectives

To assess:

- the satisfaction of caregivers with the multidisciplinary care programme
- the satisfaction of relatives with the multidisciplinary care programme
- the efficacy of a multidisciplinary care programme that includes telemonitoring, as well as all-cause mortality.

#### Design

This was a cluster randomised clinical trial with concealed allocation and analysis on an intention-to-treat basis.

#### Results

A total of 1338 patients participated in the study (573 and 765 in the experimental and control groups, respectively).

The number of days under the care of the hospital at home service per 1000 days of follow-up was significantly higher in the experimental group (2.10 vs. 0.18 days in the control group, p=0.01).

However, inpatient bed days per 1000 days of follow-up were significantly lower in the experimental group (4.4 vs. 6.86 days in the control group, p=0.019). What is more, there were significantly fewer emergency department attendances in the experimental group (120 vs. 284 in the control group, p=0.015).

Further, there were no statistically significant differences in overall mortality between the two groups (13.8% and 14.1% in the control and experimental groups, respectively), and in the experimental group, the quality of care perceived by patients and their relatives was good (measured using focus group techniques).

### Dependent patients at home

This is an ongoing before and after study with a catchment population of 68,000 people. It involves the same care pathway as the project based on geriatric patients, though with an element in primary care and without telemonitoring.

### Self-monitoring website

This is a controlled clinical trial in the recruitment phase. It will compare the EMAI intervention with self-monitoring, measuring the number admissions and emergency attendances, perceived quality of care and the number of consultations.

## Discussion

In 2010, our centre has introduced large-scale interventions with applicability to a great number of users. This has implied considering the financial sustainability of the interventions and, accordingly, we opted for low-cost organisational changes. The efficacy of these interventions has led us to extend them, with the immediate challenge of developing strategies to improve user involvement, with training programmes and protected self-care projects.

The establishment of various effective channels of communication and organisational systems makes it possible to centre care at the primary level.

The role of new technology, mainly in the area of communication and transmission of data and images, may be very important. However, it is logical to think that we will have to adapt the communication systems to the characteristics of users and centres, using various different tools according to the idiosyncrasy of each group.

## Conclusions

The controlled clinical trial of care for geriatric patients in nursing homes shows that the proposed multi-intervention may be a valid approach to decrease emergency department attendances and inpatient bed days, at the cost of a slight increase in the number of days under the care of the hospital at home service.

In general terms, the introduction of low-cost organisational changes may lead to great improvements in the care provision.

## Conference abstract Spanish

## Introducción

El hospital Donostia es el hospital de referencia de Gipuzkoa (País Vasco), con cobertura a una población de 700.000 personas.

En el año 2009 el manejo de la cronicidad se convirtió en un área estratégica del hospital dotándose en el 2011 de una estructura específica para el estudio, mejora y manejo de la cronicidad: La Unidad de Crónicos y Convalecientes-MBE (Medicina Basada en la Evidencia). El núcleo central de la asistencia de esta unidad tiene un elemento común: el manejo de los procesos en clave de MBE.

Desde el año 2005, vienen realizándose en el Hospital Donostia estudios para la identificación de reingresadores, así como intervenciones a pequeña escala (seguimiento de fallo cardiaco y EPOC) que han demostrado eficacia a la hora de disminuir los días de ingreso y visitas a urgencias, con eficiencia dudosa en el uso de la telemedicina. El siguiente paso que se planteó fue generalizar las intervenciones a la mayoría de la población.

Los objetivos que se marcaron para el 2010 fueron: poner en marcha una intervención a gran escala para los reingresadores; establecer una intervención con los centros gerontológicos de Gipuzkoa; establecer un contacto permanente con Atención Primaria; e incorporar al usuario o cuidador de forma progresiva en el manejo y control de su proceso.

## Descripción de los proyectos

A continuación se describen algunos de los estudios llevados a cabo desde la Unidad de MBE del Hospital Donostia.

### Proyecto EMAI (Estrategia Múltiple de Atención Individualizada)

#### Objetivos

- Evaluar el impacto del programa de asistencia multidisciplinar intensivo antes y después en la calidad de la asistencia percibida por el paciente frágil.
- Evaluar el impacto en el cumplimiento terapéutico.
- Evaluar el impacto en las visitas a urgencias y reingresos.

#### Metodología

Diseño: Es un estudio antes y después de una cohorte de pacientes reingresadores de fallo cardiaco o EPOC con más de un ingreso relacionado con este proceso en el año previo a la inclusión.

Población a estudio: 242 pacientes reingresadores de fallo cardiaco y EPOC en la Unidad de Crónicos-MBE del Hospital Donostia.

Descripción del EMAI: Supone la identificación del paciente frágil, marcaje informático, información personalizada, asignación de médico de referencia, asignación de una enfermera de caso, teléfono de máxima accesibilidad, contacto con el médico de familia y pacto de objetivos, consulta mensual, llamada telefónica mensual intercalada con la consulta, ingreso sin pasar por urgencias a cargo de su médico responsable.

#### Resultados

A 31 de diciembre de 2010 se analizaron 242 participantes de los cuales 132 tenían ambos procesos, existiendo 216 codificados como fallo cardiaco y 158 como EPOC.

- Reducción de los días de ingreso del 67% (3.000 días reducidos en el año de estudio).
- Reducción de la visitas a urgencias del 77%.
- Cumplimiento de medicación superior al 90%.

#### Calidad percibida

Grupos focales: Valoración muy positiva de previsión de alta, información, coordinación con centros y servicios, trato y seguimiento en consulta y telefónico.

### Ensayo clínico controlado de atención a los residentes gerontológicos

#### Objetivos Principales

- Evaluar la eficacia de un programa de asistencia multidisciplinar que incluye telemonitorización, medida como disminución del número de ingresos.
- Evaluar la pertinencia y aceptabilidad de una propuesta de pacto en torno a la planificación de los cuidados por parte de todos los implicados.

#### Objetivos Secundarios

- Evaluar la satisfacción de los cuidadores con el programa de asistencia multidisciplinar.
- Evaluar la satisfacción de los familiares con el programa de asistencia multidisciplinar.
- Evaluar la eficacia de un programa de asistencia multidisciplinar que incluye telemonitorización, medida como mortalidad por todas las causas.

#### Diseño

Ensayo clínico tipo cluster randomizado con OSA (ocultación de la secuencia de aleatorización) y AIT (análisis por intención de tratar).

#### Resultados

Han intervenido 1338 pacientes (573 en el experimental y 765 en el control).

La tasa de días de ingreso en Hospital por 1000 días de seguimiento, disminuyó en el grupo experimental (6,86 días en el grupo control frente a 4,4 días en el grupo experimental, p=0,019).

La tasa de días de ingreso en Hospitalización a Domicilio por 1000 días de seguimiento, aumentó en el grupo experimental (0,18 días en el grupo control frente a 2,10 días en el grupo experimental, p=0,01).

El número de visitas al Servicio de Urgencias disminuyó significativamente en el grupo experimental (284 visitas en el grupo control y 120 en el grupo experimental, p=0,015).

La mortalidad global no varió significativamente entre ambos grupos (13,8% en el grupo control y 14,1% en el experimental).

En el grupo experimental, la calidad asistencial percibida por pacientes y familiares fue buena (medida mediante técnica de grupos focales).

### Dependientes en domicilio

Estudio antes-después en marcha en la actualidad con una cobertura a una población de 68.000 personas. Sigue el mismo esquema de atención que el proyecto de los pacientes gerontológicos, con eje en primaria y sin telemonitorización.

### Web de autocontrol

Ensayo Clínico Controlado en fase de reclutamiento. Compara la intervención EMAI vs. autocontrol, mide ingresos, urgencias, calidad percibida, y número de consultas.

## Discusión

En nuestro centro el 2010 ha venido marcado por la implantación de intervenciones a gran escala con aplicabilidad a gran número de usuarios. Esto ha implicado decisiones sobre la sostenibilidad económica de las intervenciones, motivo por el que se ha apostado por modificaciones organizativas de bajo impacto económico. La eficacia que han demostrado estas intervenciones nos lleva a impulsarlas, planteándonos como retos inmediatos las estrategias de implicación del usuario, con programas de entrenamiento y proyectos de autocontrol protegidos.

El haber establecido canales de comunicación eficaces y sistemas organizativos distintos va a posibilitar centrar la asistencia en el nivel de atención primaria.

El papel de las nuevas tecnologías, fundamentalmente en el área de comunicación y transmisión de datos e imágenes, puede ser muy importante. Sin embargo, es lógico pensar que tengamos que adaptar los sistemas de comunicación a las características del usuario y de los centros, aplicando herramientas distintas en función de la idiosincrasia de cada uno.

## Conclusiones

El ensayo clínico controlado de atención a residentes gerontológicos muestra que la multi-intervención propuesta puede ser una alternativa válida a la hora de disminuir visitas a urgencias y días de ingreso hospitalarios, a expensas de aumentar los días de hospitalización a domicilio.

En términos generales cabe concluir que modificaciones organizativas de bajo impacto económico pueden provocar grandes mejoras en la asistencia.
